# Casein haplotypes and their association with milk production traits in Norwegian Red cattle

**DOI:** 10.1186/1297-9686-41-24

**Published:** 2009-02-20

**Authors:** Heidi Nilsen, Hanne Gro Olsen, Ben Hayes, Erling Sehested, Morten Svendsen, Torfinn Nome, Theo Meuwissen, Sigbjørn Lien

**Affiliations:** 1 Department of Animal and Aquacultural Sciences, Norwegian University of Life Sciences, Box 5003, N-1432 Aas, Norway; 2 Centre for Integrative Genetics, Norwegian University of Life Sciences, Box 5003, N-1432 Aas, Norway; 3 GENO Breeding and AI association, Norwegian University of Life Sciences, Box 5003, N-1432 Aas, Norway; 4 Animal Genetics and Genomics, Primary Industries Research Victoria, 475 Mickleham Rd, Attwood, Victoria, 3049, Australia

## Abstract

A high resolution SNP map was constructed for the bovine casein region to identify haplotype structures and study associations with milk traits in Norwegian Red cattle. Our analyses suggest separation of the casein cluster into two haplotype blocks, one consisting of the *CSN1S1*, *CSN2 *and *CSN1S2 *genes and another one consisting of the *CSN3 *gene. Highly significant associations with both protein and milk yield were found for both single SNPs and haplotypes within the *CSN1S1-CSN2-CSN1S2 *haplotype block. In contrast, no significant association was found for single SNPs or haplotypes within the *CSN3 *block. Our results point towards *CSN2 *and *CSN1S2 *as the most likely loci harbouring the underlying causative DNA variation. In our study, the most significant results were found for the SNP *CSN2_67 *with the C allele consistently associated with both higher protein and milk yields. *CSN2_67 *calls a C to an A substitution at codon 67 in β-casein gene resulting in histidine replacing proline in the amino acid sequence. This polymorphism determines the protein variants A1/B (*CSN2_67 *A allele) versus A2/A3 (*CSN2_67 *C allele). Other studies have suggested that a high consumption of A1/B milk may affect human health by increasing the risk of diabetes and heart diseases. Altogether these results argue for an increase in the frequency of the *CSN2_67 *C allele or haplotypes containing this allele in the Norwegian Red cattle population by selective breeding.

## Introduction

Several studies have reported the existence of QTL affecting milk production traits on bovine chromosome 6 (BTA6) [1,2] (summarized at http://genomes.sapac.edu.au/bovineqtl/ and http://www.vetsci.usyd.edu.au/reprogen/QTL_Map/). Two distinct regions on this chromosome affect milk traits (including protein yield, protein percentage, fat yield, fat percentage and milk yield). One QTL affecting protein and fat percentage has been positioned in a narrow region of 420 kb [3] and a putative functional polymorphism in the *ABCG2 *gene underlying the QTL has been suggested [4,5]. The second region on BTA6 associated with milk traits maps to the casein cluster [*e.g*. [6-11]]. The casein cluster is composed of four genes; α_s1_-, β-, α_s2_- and κ-casein (*CSN1S1*, *CSN2*, *CSN1S2 *and *CSN3*, respectively) producing approximately 80 percent of the protein content of cow's milk [12]. The four casein genes have been mapped in the order *CSN1S1-CSN2-CSN1S2-CSN3 *to bovine chromosome 6 (BTA6) at q31-33 by *in situ *hybridisation [13,14].

Several polymorphisms have been detected in the open reading frame (reviewed by [12]) and in noncoding regions such as the 5'-flanking region of the casein genes [15,16]. The most common genetic variants in western dairy breeds are α_*s*1_-casein B (here denoted CSN1S1_192*A) and C (CSN1S1_192*G), β-casein A1 (CSN2_67*A), A2 (CSN2_67*C) and B (CSN2_122*C), and κ-casein A (CSN3_136*C), B (CSN3_136*T) and E (CSN3_155*G).

In the present study, we have constructed a dense SNP map in the casein region. The map facilitates accurate haplotype construction and was used for comprehensive association studies in Norwegian Red cattle.

## Methods

### Animals in the QTL study

All animals in the study belonged to the Norwegian Red cattle breed. For the chromosome wide QTL scan, animals were organized in a granddaughter design consisting of 18 elite sire families with a total of 716 sons and 507,000 granddaughters. To fine-map QTL in the casein region, the animal data was expanded to 31 elite sire families with a total of 1112 sons, ranging from 23 to 70 sons for the smallest and largest families, respectively. The total number of daughters in this analysis was approximately 1.9 million, with an average of 1670 daughters per son. The families were chosen based on sufficiently large family sizes and/or availability of trait data. The pedigree of each animal in the study was traced back as far as known. Daughter yield deviations (DYDs) of the sons were used as performance information in the analyses. The DYDs for milk production traits [protein percentage (P%), protein yield (PY), milk yield (MY), fat percentage (F%) and fat yield (FY)] were available from the national genetic evaluation carried out by GENO Breeding and AI Association, and evaluated using a BLUP animal model [17].

### Marker map

For the initial QTL scan, we used a map consisting of 399 SNPs covering the entire BTA6 [18]. To fine-map QTL, we constructed a dense marker map consisting of 73 SNPs in and around the casein region on BTA6, covering approximately 750 kb. Fifty-four of the 73 SNPs in the map were detected by PCR resequencing of promoters and exon regions of all four casein genes (*CSN1S1*, *CSN2*, *CSN1S2 *and *CSN3*), nine SNPs were available from [19], whereas ten SNPs were selected from the Bovine Genome Sequencing Project [20]. Physical distances between markers were determined from one single scaffold, NW_001495211, available from the latest assembly of the bovine genome Btau_4.0 [20]. The average distance between SNPs was 10,462 bp (ranging from 7 to 302,143 bp). A description of the SNPs, including accession numbers in dbSNP, assays for genotyping on the MassARRAY system (Sequenom, San Diego, USA), marker allele frequencies and predicted physical distances between markers can be found in Additional file 1.

### QTL analysis

A combined linkage and linkage disequilibrium (LDLA) method [5] was used to analyze milk production traits based on the information on markers from the 399-marker map described in [18] and a dense SNP map (73 markers) constructed for the casein region (see Additional file 1). For the midpoint of each marker bracket, the log-likelihood of a model containing the QTL (LogL(**G_i_**)) was calculated as well as a model fitting only background genes (LogL(0)) using the ASREML package [21]. Our test statistic, LogL difference, was then calculated as the difference in log-likelihood between the first and the second model. This LogL difference times 2 is equal to the Likelihood Ratio Test-statistic (LRT) of [22]. According to Baret and coworkers, the distribution of the LRT under the null hypothesis can be seen as a mixture of two chi square distributions with 0 and 1 degree of freedom (df), respectively. Significance levels for the LRT are then found from a chi square distribution with 1 df but doubling the probability levels [22]. Then, to obtain a significance level of 0.0005, the LRT value corresponding to a chi square distribution with 1 df and P = 0.001 is utilized. This LRT value is 10.8, and thus the corresponding LogL difference must be 5.4 or higher to achieve a significance level of 0.0005.

### SNP association tests

DYDs of the sons were used as performance information in the analyses. The model fitted to the performance information for each trait and each SNP was: *DYD*_*i *_= *μ *+ *s*_*i *_+ *x*_*i*_*b *+ *a*_*i *_+ *e*_*i *_where DYD_i _is performance of son i, μ is the overall mean, s_i _is a fixed effect of sire of son i, x_i _is 0 if son i is homozygous 1 1 (*e.g*. AA); 1 if son i is heterozygous 1 2 (*e.g*. AT or TA); or 2 if son i is homozygous 2 2 (*e.g*. TT), b is the effect of the SNP, a_i _is a polygenic effect of son i, and e_i _is a residual effect. For each single marker, the log-likelihood of a model containing the SNP effect (LogL(H1)) was calculated as well as a model without this SNP effect (LogL(H0)) using the ASREML package [21]. Our test statistic, LogL difference, was then calculated as the difference in LogL between the first and the second model as described above. A SNP effect was regarded significant if the LogL difference exceeded 5.4.

Additionally, multiple SNP association tests were carried out for the most significant markers from the single SNP association test. The tests were implemented by fitting a fixed effect of the SNP in the above-mentioned model and repeating the analyses for the most significant SNPs in turn. Test statistics for the analyses were as described above.

### LD and haplotype block structure of the casein region

An analysis package, CRIHAP, was developed for determining haplotypic phases and imputing missing genotypes for all individuals (Nome and Lien, unpublished). The programs are based on both linkage and linkage disequilibrium information generated by the CRI-MAP 2.4 [23] and PHASE version 2.1 [24,25] programs. Map information and genotypes for all animals were imported into the Haploview program [26] to calculate LD (r^2^) between markers.

### Haplotype analysis

Haplotype blocks were constructed for the casein loci *CSN1S1*, *CSN2 *and *CSN1S2 *for which we found highly significant brackets or single SNPs associated with protein yield. A script was made to deduce maternal and paternal haplotypes for all individuals and different haplotype blocks using haplotypic phases from the CRIHAP program package. As for the single SNP analyses, DYDs of the sons were used as performance information in the analyses. The model fitted to the DYDs, for each trait and each haplotype, was *DYD*_*i *_= *μ *+ *s*_*i *_+ *x*_*i*_*b *+ *a*_*i *_+ *e*_*i *_where DYD_i _is the performance of son i, μ is the overall mean, s_i _is a fixed effect of sire of son i, x_i _is a row-vector indicating which haplotypes and how many copies are carried by the son; and b is a column indicating the random effects of the haplotypes; a_i _is a random polygenic effect of son i, and e_i _is a residual effect. The test statistic (LogL difference) was found as previously described for the single SNP association test. Phenotypic standard deviations for protein and milk yield were 36.75 kg and 1137.79 kg, respectively. These deviations were used to scale the haplotype effects into phenotypic standard deviations for each of the traits for a standardised presentation.

## Results

### Chromosome wide QTL scan

**Figure 1:**
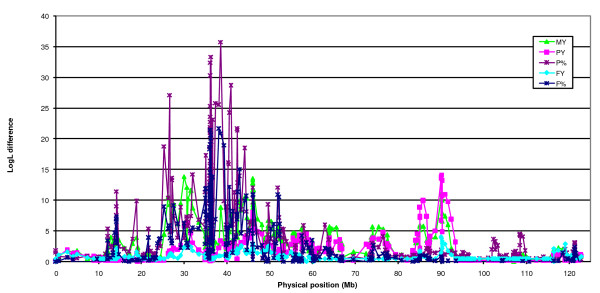
**LDLA QTL analysis for milk yield (MY), protein yield (PY), protein percentage (P%), fat yield (FY), and fat percentage (F%) using the 399-marker map of Nilsen *et al***. [18]. Points illustrate bracket midpoints; the physical distance is scaled in Mb and the y-axis denotes the LogL differences.

**Figure 2:**
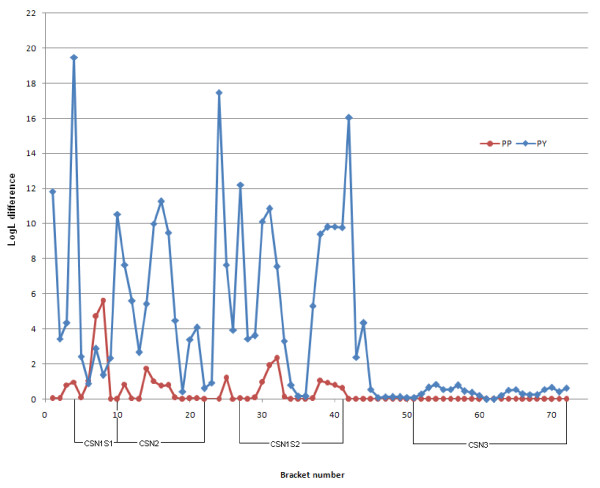
**LDLA QTL analysis for protein percentage (P%) and protein yield (PY) in the interval between marker BTA6-107923 and BTA6-09701 (markers in NW_001495211)**. For better readability, the x-axis has been presented as bracket numbers where points illustrate bracket midpoints; the y-axis reflects the LogL differences.

Results of the initial QTL scan for milk yield, protein yield, protein percentage, fat yield and fat percentage (LDLA analysis using the 399-marker map) are shown in Figure 1. For details about the markers, see Table S1 in Nilsen *et al*. [18] or http://cilit.umb.no/maps/. The analysis reveals highly significant results (LogL difference > 5.4, P < 0.0005) mainly in two different regions. Milk yield, protein yield and especially fat and protein percentages show highly significant results in the region between approximately 25 and 45 Mb. This QTL, previously fine-mapped in Norwegian Red cattle [3], is potentially caused by a polymorphism in the *ABCG2 *gene [4,5]. Additionally, highly significant results were found for milk and protein yields in the casein cluster region at approximately 90 Mb. The results from the initial scan were followed up by LDLA analyses in a high-resolution map constructed for the casein region (73 SNPs) and using an extended number of families. The result of this analysis for protein yield and percentage are shown in Figure 2 (for details about the markers, see Additional file 1). The LogL difference for protein yield was found for the interval between the markers *BTA6-02720 *and *CSN1S1-Prom_175 *(LogL difference = 19.5), but several additional significant results appear for numerous marker brackets in *CSN2 *and *CSN1S2*. No significant result was found for marker brackets in the *CSN3 *gene. The interval between *CSN1S1_192 *and *CSN1S1-BMC_17969 *was the only one with significant LogL difference for protein percentage (LogL difference = 5.6).

### SNP association tests

**Figure 3:**
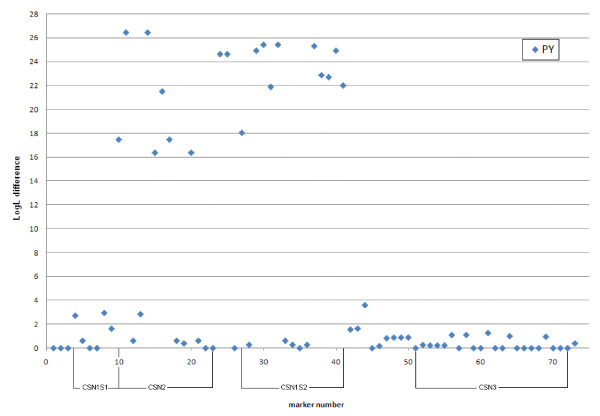
**Single SNP association test results for protein yield**. The x-axis denotes marker number and the y-axis the LogL differences.

**Figure 4:**
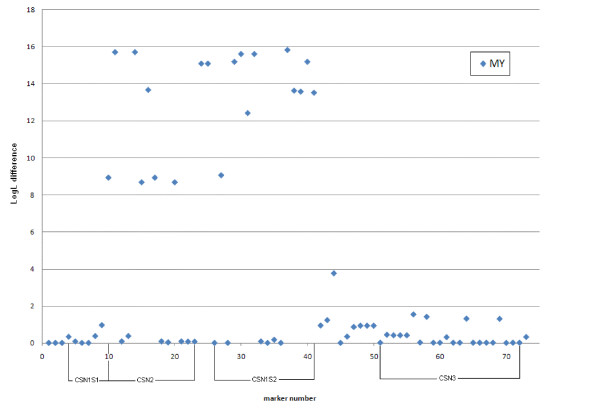
**Single SNP association test results for milk yield**. The x-axis denotes marker number and the y-axis the LogL differences.

Data was also analysed for association between single SNPs and DYDs for protein yield and milk yield. Highly significant results were found for a number of SNPs in *CSN2 *and *CSN1S2 *for both protein yield (PY) and milk yield (MY) (Figure 3 and Figure 4, respectively). SNPs with the highest LogL differences were *CSN2-BMC_9215 *and *CSN2_67 *for both traits (LogL difference = 26.4 for PY and 15.7 for MY for both SNPs), in addition to *CSN1S2-BMC_17192 *for MY (LogL difference = 15.8).

**Figure 5:**
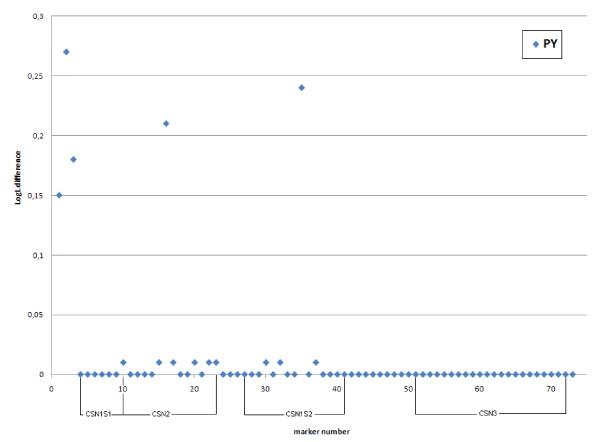
**A multiple SNP association test results for protein yield when fitting *CSN2_67 *as fixed effect in the model**. The x-axis denotes marker number and the y-axis the LogL differences.

In most cases when fitting an effect of the most significant SNPs in a multiple SNP association test it highly reduced LogL differences for the other SNPs in the region. The most striking results were found for SNPs *CSN2-BMC_9215 *and *CSN2_67*. These two SNPs are in complete LD with each other and both removed almost all peaks for other markers in the region. The result for *CSN_67 *is presented in Figure 5. In accordance with the LDLA results no significant association was found between SNPs in the *CSN3 *gene and DYDs for PY.

### Extent of LD and haplotype reconstruction

**Figure 6:**
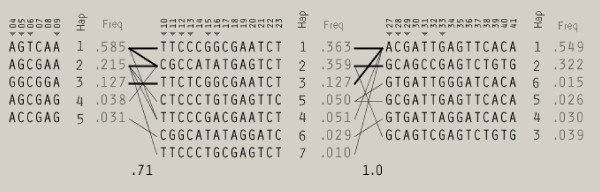
**Loci haplotype combinations; *CSN1S1 *(marker 4 to 9), *CSN2 *(marker 10 to 23) and *CSN1S2 *(marker 27 to 41), and their haplotype number (Hap; black numbers) and frequencies (Freq; grey numbers) in 1143 Norwegian Red bulls (sires and sons)**. TagSNPs for each haplotype block, identified by pairwise tagging in the Haploview program, are presented by triangles in the figure; more marker information can be found in Additional file 1.

**Figure 7:**
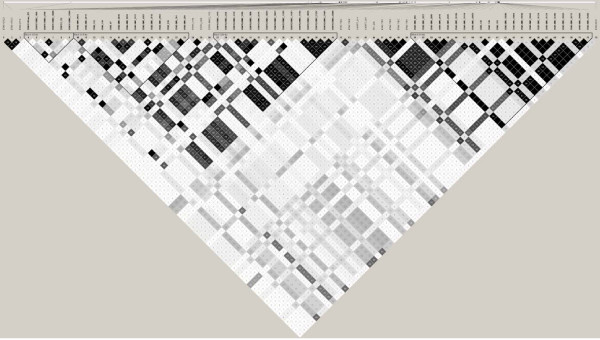
**LD across the casein segment visualized using the Haploview program **[26]. Each diamond contains the level of LD measured by r^2 ^between the markers specified; darker tones correspond to increasing levels of r^2^; triangles indicate division by loci.

The dense SNP map in the casein region made it possible to construct haplotypes within the casein loci. Such an analysis revealed five haplotypes for *CSN1S1*, seven haplotypes for *CSN2 *and six haplotypes for *CSN1S2 *(Figure 6). LD between pairs of loci varied from complete disequilibrium to almost no disequilibrium, and was much higher between SNPs in *CSN2 *and *CSN1S2 *than between SNPs in any other gene (Figure 7). The extent of LD between SNPs within *CSN1S1*, *CSN2 *and *CSN1S2 *allowed us to construct an extended haplotype block covering all three genes, creating 12 haplotypes with a population frequency above 0.9% (Additional file 2).

### Haplotype effects

**Figure 8:**
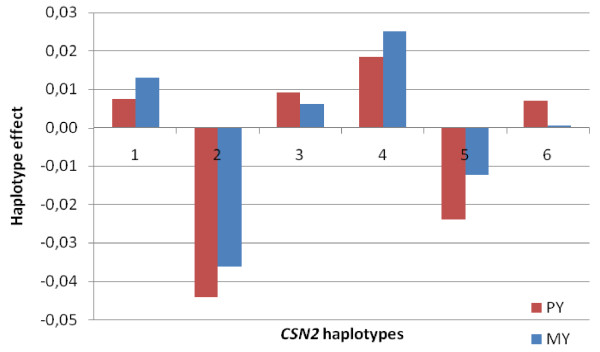
**Effects of *CSN2 *(β-casein) haplotypes on PY and MY. The x-axis denotes haplotype number and the y-axis shows haplotype effects in phenotypic standard deviations of the traits**. Significance levels of haplotype effects are given in Table 1.

**Figure 9:**
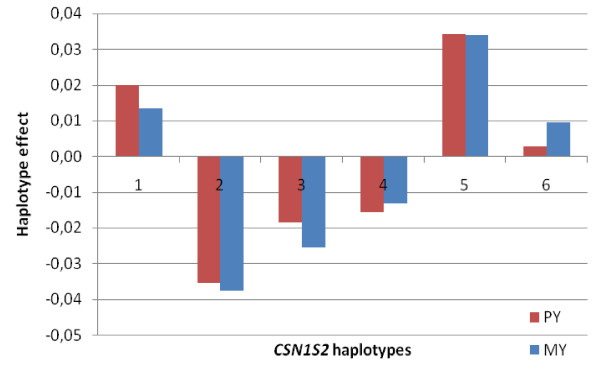
**Effect of *CSN1S2 *(α_s2_-casein) haplotypes on PY and MY. The x-axis denotes haplotype number and the y-axis shows haplotype effects in phenotypic standard deviations of the traits**. Significance levels of haplotype effects are given in Table 1.

**Figure 10:**
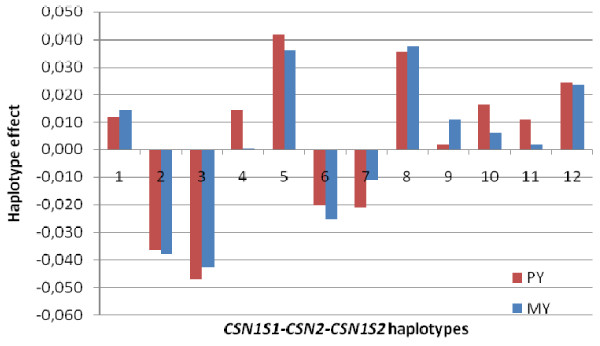
**Haplotype effects on PY and MY for a haplotype block constructed for *CSN1S1*-*CSN2*-*CSN1S2***. Only haplotypes with population frequency above 0.9% are shown; the x-axis denotes haplotype number and the y-axis shows haplotype effects given in phenotypic standard deviations of the traits; significance levels of haplotype effects are given in Table 1.

LogL differences for the four individual casein loci for PY and MY are shown in Table 1. As shown in Figure 8 and Figure 9, respectively, highly significant results were found in the *CSN2 *and *CSN1S2 *genes for both PY and MY. Six haplotypes were identified for *CSN2*. Estimation of the effect of haplotypes within loci on PY and MY revealed two haplotypes that tend to be negative (haplotype 2 and 5) and four haplotypes that tend to be positive (haplotypes 1, 3, 4 and 6) for *CSN2 *(Figure 8). For *CSN1S2*, we detected three haplotypes that are negative for both MY and PY (haplotypes 2, 3 and 4) (Figure 9). In contrast, both haplotypes 1 and 5 seem to be positive for both MY and PY. In addition, LogL differences for the extended haplotype block covering *CSN1S1-CSN2-CSN1S2 *were highly significant for both PY and MY (Table 1). The effects of the 12 haplotypes created for this block are shown in Figure 10. Effects of haplotypes for MY and PY were in the same direction for both traits, with four haplotypes tending to be negative (haplotypes 2, 3, 6 and 7) and eight haplotypes that seem to be positive for both traits.

**Table 1:** Level of significance of haplotype effects within locus/haplotype block for protein yield (PY) and milk yield (MY). LogL differences above 5.4 are regarded as significant (P < 0.0005)

Haplotype	LogL differences	
	**Protein yield**	**Milk yield**
*CSN1S1*	2.7	0.2
*CSN2*	22.7	13.0
*CSN1S2*	24.1	14.4
*CSN3*	3.0	2.4
*CSN1S1-CSN2-CSN1S2*	22.1	13.3

## Discussion

Our analysis of a dense SNP map in the casein region using the LDLA methodology revealed a high number of significant marker brackets for protein yield especially in *CSN2 *and *CSN1S2 *(Figure 1 and Figure 2). The fact that LDLA could not pin point a single marker bracket harbouring the QTL can probably be explained by a high degree of LD between the markers in the region. Analysis of the extent of LD in the region showed high LD in two segments (one segment consisting of *CSN1S1*, *CSN2 *and *CSN1S2 *and another one consisting of *CSN3*) (Figure 7). The two segments seem to be broken by a possible recombinant hotspot. Nilsen *et al*. [27] have reported evidence for a recombination hotspot between *CSN1S2 *and *CSN3*, confirming these findings. Hayes *et al*. [28] have also reported a recombination hotspot in the casein region in goat. Despite the fact that all four casein genes are coordinately expressed at high levels in a tissue- and stage-specific fashion, the κ-casein gene is not evolutionarily related to the three other casein genes (α_s1_, β and α_s2_) [29]. The calcium-sensitive caseins (α_s1_, β and α_s2_) have originated from a common ancestral gene via intergenic and intragenic duplications [30] and share common regulatory motifs [31], whereas it has been suggested that the κ-casein is related to fibrinogens on the basis of amino acid sequence similarities [32]. This evolutionary origin may also account for the LD segmentation described in this paper.

In accordance with the LDLA results, the single SNP association tests did not detect significant results for the *CSN3 *region, whereas a large number of significant associations were detected between SNPs within *CSN2 *and *CSN1S2*, and protein and milk yields. The most significant results were found for *CSN2_67*, *CSN2-BMC_9215 *and *CSN1S2-BMC_17192*. When fitting *CSN2_67 *as fixed effect in a multiple SNP association test it removed almost all peaks for other markers in the region (Figure 5). This indicates that *CSN2_67 *is in strong LD with the underlying causal variation in Norwegian Red. However, the fact that the two SNP alleles seem to display contradictory effects in various cattle breeds [6-8,10] argue against *CSN2_67 *as being an underlying causal variation.

Notably, *CSN2_67 *determines the genetic variants A1/B versus A2. The C → A substitution at codon 67 results in the exchange of proline with histidine in the amino acid sequence [33], leading to a difference in the conformation of the secondary structure of the expressed protein. It is thought that the A allele at *CSN2_67 *yields the bioactive peptide beta-casomorphin 7 (*BCM-7*), a peptide with opioid-like effect, which may play an unclear role in the development of some human diseases (for a review, see [34]). It has been suggested that a high consumption of A1/B milk increases the risk of type 1 (insulin-dependent) diabetes mellitus [35], ischaemic heart disease [36], sudden infant death syndrome (SIDS) [37], the aggravation of symptoms associated with schizophrenia and autism (reviewed in [38]), and may also correlate with milk allergy [39,40] in humans.

The high degree of LD between SNPs allowed us to construct haplotypes within and across the *CSN1S1, CSN2 *and *CSN1S2 *genes and investigate associations between haplotypes and DYDs for protein yield and milk yield. Analysis for *CSN2 *reveals two haplotypes (2 and 5) that associate with low protein yield values whereas four haplotypes (1, 3, 4 and 6) seem to be associated with higher PY levels (Figure 8). The difference between these two classes of haplotypes is characterized by the three SNPs *CSN2-BMC_9215*, *CSN2_67 *and *CSN2-BMC_6334 *(marker 11, 14 and 16, respectively; Figure 6), all of which have high LogL differences in the single SNP association test for both PY and MY.

For the *CSN1S2 *locus, we detected two haplotypes that seem to be associated with increased protein yield (1 and 5) whereas three haplotypes (2, 3 and 4) tend to be associated with a lower protein yield (Figure 9). *CSN1S2 *haplotype 5 is part of *CSN2 *haplotype 5 (see Figure 6). No significant haplotype was detected for *CSN1S1 *(data not shown). The main reason is probably that *CSN2 *haplotypes 1 (positive for protein yield) and 2 (negative for protein yield) combine into one frequent haplotype in *CSN1S1*.

For the extended block covering *CSN1S1-CSN2-CSN1S2*, we detected four haplotypes that associate with reduced milk and protein production (haplotype 2, 3, 6 and 7). Interestingly, all of these haplotypes contain the A-allele of *CSN2_67 *(the A1/B variant), in addition to the G-allele of *CSN2-BMC_9215 *(Additional file 2). In contrast, haplotypes containing the *CSN2-A2 *variant tend to associate with increased milk and protein yields. As consumption of CSN-A2 milk may have an accompanying positive effect on human health [39,40,35,34,38,37] it is recommended to increase the frequency of this allele in the Norwegian cattle population. One possible way of implementation would be to preselect calves prior to phenotype testing for growth performance and progeny testing for milk performance.

## Competing interests

The authors declare that they have no competing interests.

## Authors' contributions

HN participated in designing the study, carried out the SNP detection, was involved in the construction of the map and the haplotypes, and drafted the manuscript. HGO performed the QTL analysis, single SNP and haplotype association tests, and helped to draft the manuscript. BH participated in supervising the study. ES and MS provided all pedigree and performance information. TN designed the CRIHAP program script. TM participated in the statistical analysis. SL supervised the study, coordinated the SNP identification and genotyping process, constructed the map, performed the haplotype construction, was involved in the design of the CRIHAP program script, and finalized the manuscript. All authors read and approved the final manuscript.

## Supplementary Material

Additional file 1 **Detailed SNP information**. A list of the SNPs, including accession numbers in dbSNP, assays for genotyping on the MassARRAY system (Sequenom, San Diego, USA), marker allele frequencies, predicted physical distances between markers, and surrounding sequence of each SNP. Click here for file

Additional file 2 Haplotypes covering the *CSN1S1*-*CSN2*-*CSN1S2 *region (marker 4 to 41), their haplotype number and frequencies in 1143 Norwegian Red bulls (sires and sons) Click here for file

## References

[B1] Khatkar MS Thomson PC Tammen I Raadsma HW Quantitative trait loci mapping in dairy cattle: review and meta-analysis Genet Sel Evol 2004 36 163 190 10.1051/gse:2003057 15040897 PMC2697184

[B2] Smaragdov MG Genetic mapping of loci responsible for milk quality parameters in dairy cattle Genetika 2006 42 5 21 16523661

[B3] Olsen HG Lien S Gautier M Nilsen H Roseth A Berg PR Sundsaasen KK Svendsen M Meuwissen THE Mapping of a milk production quantitative trait locus to a 420-kb region on bovine chromosome 6 Genetics 2005 169 275 283 15466433 10.1534/genetics.104.031559 PMC1448861

[B4] Cohen-Zinder M Seroussi E Larkin DM Loor JJ Everts-van der Wind A Lee JH Drackley JK Band MR Hernandez AG Shani M Lewin HA Weller JI Ron M Identification of a missense mutation in the bovine ABCG2 gene with a major effect on the QTL on chromosome 6 affecting milk yield and composition in Holstein cattle Genome Res 2005 15 936 944 15998908 10.1101/gr.3806705 PMC1172037

[B5] Olsen HG Nilsen H Hayes B Berg PR Svendsen M Lien S Meuwissen T Genetic support for a quantitative trait nucleotide in the ABCG2 gene affecting milk composition of dairy cattle BMC Genetics 2007 8 32 17584938 10.1186/1471-2156-8-32 PMC1924865

[B6] Boettcher PJ Caroli A Stella A Chessa S Budelli E Canavesi F Ghiroldi S Pagnacco G Effects of casein haplotypes on milk production traits in Italian Holstein and Brown Swiss cattle J Dairy Sci 2004 87 4311 4317 15545395 10.3168/jds.S0022-0302(04)73576-6

[B7] Bovenhuis H Weller JI Mapping and analysis of dairy cattle quantitative trait loci by maximum likelihood methodology using milk protein genes as genetic markers Genetics 1994 137 267 280 8056316 10.1093/genetics/137.1.267 PMC1205943

[B8] Ikonen T Bovenhuis H Ojala M Ruottinen O Georges M Associations between casein haplotypes and first lactation milk production traits in Finnish Ayrshire cows J Dairy Sci 2001 84 507 514 11233036 10.3168/jds.S0022-0302(01)74501-8

[B9] Lien S Gomez-Raya L Steine T Fimland E Rogne S Associations between casein haplotypes and milk yield traits J Dairy Sci 1995 78 2047 2056 8550914 10.3168/jds.S0022-0302(95)76830-8

[B10] Velmala R Vilkki J Elo K Maki-Tanila A Casein haplotypes and their association with milk production traits in the Finnish Ayrshire cattle Anim Genet 1995 26 419 425 8572365 10.1111/j.1365-2052.1995.tb02694.x

[B11] Velmala RJ Vilkki HJ Elo KT de Koning DJ Maki-Tanila AV A search for quantitative trait loci for milk production traits on chromosome 6 in Finnish Ayrshire cattle Anim Genet 1999 30 136 143 10.1046/j.1365-2052.1999.00435.x 10376304

[B12] Farrell HM Jr Jimenez-Flores R Bleck GT Brown EM Butler JE Creamer LK Hicks CL Hollar CM Ng-Kwai-Hang KF Swaisgood HE Nomenclature of the proteins of cows' milk – sixth revision J Dairy Sci 2004 87 1641 1674 15453478 10.3168/jds.S0022-0302(04)73319-6

[B13] Ferretti L Leone P Sgaramella V Long range restriction analysis of the bovine casein genes Nucleic Acids Res 1990 18 6829 6833 2263448 10.1093/nar/18.23.6829 PMC332738

[B14] Threadgill DW Womack JE Genomic analysis of the major bovine milk protein genes Nucleic Acids Res 1990 18 6935 6942 1979856 10.1093/nar/18.23.6935 PMC332753

[B15] Martin P Szymanowska M Zwierzchowski L Leroux C The impact of genetic polymorphisms on the protein composition of ruminant milks Reprod Nutr Dev 2002 42 433 459 10.1051/rnd:2002036 12537255

[B16] Schild TA Geldermann H Variants within the 5'-flanking regions of bovine milk-protein-encoding genes. III. Genes encoding the Ca-sensitive caseins α_s1_, α_s2 _and β Theor Appl Genet 1996 93 887 893 10.1007/BF00224090 24162422

[B17] Svendsen M Heringstad B New genetic evaluation for clinical mastitis in multiparous Norwegian Red cows Interbull Bull 2006 35 8 11

[B18] Nilsen H Hayes B Berg PR Roseth A Sundsaasen KK Nilsen K Lien S Construction of a dense SNP map for bovine chromosome 6 to assist the assembly of the bovine genome sequence Anim Genet 2008 39 97 104 10.1111/j.1365-2052.2007.01686.x 18307581

[B19] Lien S Rogne S Bovine casein haplotypes: number, frequencies and applicability as genetic markers Anim Genet 1993 24 373 376 8291739 10.1111/j.1365-2052.1993.tb00343.x

[B20] Bovine Genome Project http://www.hgsc.bcm.tmc.edu/

[B21] Gilmour AR Cullis BR Welham SJ Thompson R ASREML reference manual 2001 New South Wales Agriculture

[B22] Baret PV Knott SA Visscher PM On the use of linear regression and maximum likelihood for QTL mapping in half-sib designs Genet Res 1998 72 149 158 10.1017/S0016672398003450 9883097

[B23] Green P Falls K Crooks S Documentation for CRI-MAP, version 2.4 1990 Washington University School of Medicine St. Louis

[B24] Stephens M Donnelly P A comparison of bayesian methods for haplotype reconstruction from population genotype data Am J Hum Genet 2003 73 1162 1169 14574645 10.1086/379378 PMC1180495

[B25] Stephens M Smith NJ Donnelly P A new statistical method for haplotype reconstruction from population data Am J Hum Genet 2001 68 978 989 11254454 10.1086/319501 PMC1275651

[B26] Barrett JC Fry B Maller J Daly MJ Haploview: analysis and visualization of LD and haplotype maps Bioinformatics 2005 21 263 265 10.1093/bioinformatics/bth457 15297300

[B27] Nilsen H Olsen HG Hayes B Nome T Svendsen M Meuwissen T Lien S Identification of a haplotype on bovine chromosome 6 reducing clinical mastitis while simultaneously increasing protein yield Anim Genet in press

[B28] Hayes B Hagesaether N Adnoy T Pellerud G Berg PR Lien S Effects on production traits of haplotypes among casein genes in Norwegian goats and evidence for a site of preferential recombination Genetics 2006 174 455 464 16849606 10.1534/genetics.106.058966 PMC1569804

[B29] Alexander LJ Stewart AF Mackinlay AG Kapelinskaya TV Tkach TM Gorodetsky SI Isolation and characterization of the bovine kappa-casein gene Eur J Biochem 1988 178 395 401 10.1111/j.1432-1033.1988.tb14463.x 3208764

[B30] Groenen MA Dijkhof RJ Verstege AJ Poel JJ van der The complete sequence of the gene encoding bovine alpha s2-casein Gene 1993 123 187 193 10.1016/0378-1119(93)90123-K 8428658

[B31] Groenen MA Dijkhof RJ Poel JJ van der van Diggelen R Verstege E Multiple octamer binding sites in the promoter region of the bovine alpha s2-casein gene Nucleic Acids Res 1992 20 4311 4318 1508722 10.1093/nar/20.16.4311 PMC334141

[B32] Jolles P Loucheux-Lefebvre MH Henschen A Structural relatedness of kappa-casein and fibrinogen gamma-chain J Mol Evol 1978 11 271 277 10.1007/BF01733837 722804

[B33] Groves ML Some minor components of casein and other phosphoproteins in milk. A review J Dairy Sci 1969 52 1155 1165

[B34] Kaminski S Cieslinska A Kostyra E Polymorphism of bovine beta-casein and its potential effect on human health J Appl Genet 2007 48 189 198 17666771 10.1007/BF03195213

[B35] Elliott RB Harris DP Hill JP Bibby NJ Wasmuth HE Type I (insulin-dependent) diabetes mellitus and cow milk: casein variant consumption Diabetologia 1999 42 292 296 10.1007/s001250051153 10096780

[B36] McLachlan CN Beta-casein A1, ischaemic heart disease mortality, and other illnesses Med Hypotheses 2001 56 262 272 10.1054/mehy.2000.1265 11425301

[B37] Sun Z Zhang Z Wang X Cade R Elmir Z Fregly M Relation of beta-casomorphin to apnea in sudden infant death syndrome Peptides 2003 24 937 943 10.1016/S0196-9781(03)00156-6 12948848

[B38] Knivsberg AM Reichelt KL Nodland M Reports on dietary intervention in autistic disorders Nutr Neurosci 2001 4 25 37 11842874 10.1080/1028415x.2001.11747348

[B39] Chatchatee P Jarvinen KM Bardina L Beyer K Sampson HA Identification of IgE- and IgG-binding epitopes on alpha(s1)-casein: differences in patients with persistent and transient cow's milk allergy J Allergy Clin Immunol 2001 107 379 383 10.1067/mai.2001.112372 11174208

[B40] Chatchatee P Jarvinen KM Bardina L Vila L Beyer K Sampson HA Identification of IgE and IgG binding epitopes on beta- and kappa-casein in cow's milk allergic patients Clin Exp Allergy 2001 31 1256 1262 10.1046/j.1365-2222.2001.01167.x 11529896

