# Supplementary material for: Casein haplotypes and their association with milk production traits in Norwegian Red cattle
Source: Genet Sel Evol. 2009 Feb 20;41(1):24. doi: 10.1186/1297-9686-41-24 (PMC3225817; doi:10.1186/1297-9686-41-24)
Supplement: Additional file 2 — Haplotypes covering the CSN1S1-CSN2-CSN1S2 region (marker 4 to 41), their haplotype number and frequencies in 1143 Norwegian Red bulls (sires and sons) [file 1297-9686-41-24-S2.doc]

| **Haplotypes** | **CSN1S1-Prom_175** | **CSN1S1-BMC_1995** | **CSN1S1-BMC_5798** | **CSN1S1-BMC_16680** | **CSN1S1_192** | **CSN1S1-BMC_17969** | **CSN2-BMC_9472** | **CSN2-BMC_9215** | **CSN2_122** | **CSN2_110** | **CSN2_67** | **CSN2-BMC_6640** | **CSN2-BMC_6334** | **CSN2-BMC_5775** | **CSN2-BMC_4393** | **CSN2-BMC_3118** | **CSN2-BMC_2364** | **CSN2_516** | **CSN2-BMC_344** | **CSN2-BMC_269** | **STATH_1754** | **STATH_2068** | **STATH-BMC2_1578** | **CSN1S2-BMC_1915** | **CSN1S2-BMC_2544** | **CSN1S2-BMC_3312** | **CSN1S2-BMC_5105** | **CSN1S2-BMC_7008** | **CSN1S2-BMC_8316** | **CSN1S2-BMC_10215** | **CSN1S2-BMC_10431** | **CSN1S2-BMC_11255** | **CSN1S2-BMC_14361** | **CSN1S2-BMC_17192** | **CSN1S2-BMC_18035** | **CSN1S2-BMC_21257** | **CSN1S2-BMC_21965** | **CSN1S2-BMC_22088** | **Relative frequency** |
| --- | --- | --- | --- | --- | --- | --- | --- | --- | --- | --- | --- | --- | --- | --- | --- | --- | --- | --- | --- | --- | --- | --- | --- | --- | --- | --- | --- | --- | --- | --- | --- | --- | --- | --- | --- | --- | --- | --- | --- |
|  | 4 | 5 | 6 | 7 | 8 | 9 | 10 | 11 | 12 | 13 | 14 | 15 | 16 | 17 | 18 | 19 | 20 | 21 | 22 | 23 | 24 | 25 | 26 | 27 | 28 | 29 | 30 | 31 | 32 | 33 | 34 | 35 | 36 | 37 | 38 | 39 | 40 | 41 |  |
| **1** | A | G | T | C | A | A | T | T | C | C | C | G | G | C | G | A | A | T | C | T | A | T | C | A | C | G | A | T | T | G | A | G | T | T | C | A | C | A | 0.355 |
| **2** | A | G | T | C | A | A | C | G | C | C | A | T | A | T | G | A | G | T | C | T | G | C | C | G | C | A | G | C | C | G | A | G | T | C | T | G | T | G | 0.186 |
| **3** | A | G | C | G | A | A | C | G | C | C | A | T | A | T | G | A | G | T | C | T | G | C | C | G | C | A | G | C | C | G | A | G | T | C | T | G | T | G | 0.127 |
| **4** | G | G | C | G | G | A | T | T | C | T | C | G | G | C | G | A | A | T | C | T | A | T | C | A | C | G | A | T | T | G | A | G | T | T | C | A | C | A | 0.126 |
| **5** | A | G | T | C | A | A | T | T | C | C | C | G | A | C | G | A | A | T | C | T | A | T | C | A | C | G | A | T | T | G | A | G | T | T | C | A | C | A | 0.037 |
| **6** | A | G | C | G | A | G | C | G | C | C | A | T | A | T | G | A | G | T | C | T | G | C | C | G | C | A | G | T | C | G | A | G | T | C | T | G | T | G | 0.037 |
| **7** | A | C | C | G | A | G | C | G | G | C | A | T | A | T | A | G | G | A | T | C | A | T | T | G | T | G | A | T | T | A | G | G | A | T | C | A | C | A | 0.029 |
| **8** | A | G | C | G | A | A | C | T | C | C | C | T | G | T | G | A | G | T | T | C | A | T | T | G | C | G | A | T | T | G | A | G | T | T | C | A | C | A | 0.026 |
| **9** | A | G | C | G | A | A | C | T | C | C | C | T | G | T | G | A | G | T | T | C | A | T | T | G | T | G | A | T | T | G | G | G | A | T | C | A | C | A | 0.014 |
| **10** | A | G | C | G | A | A | T | T | C | C | C | G | A | C | G | A | A | T | C | T | A | T | C | A | C | G | A | T | T | G | A | G | T | T | C | A | C | A | 0.013 |
| **11** | A | G | C | G | A | A | T | T | C | C | C | T | G | C | G | A | G | T | C | T | A | T | C | A | C | G | A | T | T | G | A | G | T | T | C | A | C | A | 0.010 |
| **12** | A | G | C | G | A | A | C | T | C | C | C | T | G | T | G | A | G | T | T | C | A | T | C | G | C | G | A | T | T | G | A | G | T | T | T | G | C | G | 0.009 |

Haplotypes covering the *CSN1S1*-*CSN2*-*CSN1S2* region (marker 4 to 41), their haplotype number and

frequencies in 1143 Norwegian Red bulls (sires and sons)

[More marker information can be found in Additional file 1]
